# Structural Analysis of *Staphylococcus aureus* Serine/Threonine Kinase PknB

**DOI:** 10.1371/journal.pone.0039136

**Published:** 2012-06-11

**Authors:** Sonja Rakette, Stefanie Donat, Knut Ohlsen, Thilo Stehle

**Affiliations:** 1 Interfaculty Institute of Biochemistry, University of Tübingen, Tübingen, Germany; 2 Institute for Molecular Infection Biology, University of Würzburg, Würzburg, Germany; 3 Department of Pediatrics, Vanderbilt University School of Medicine, Nashville, Tennessee, United States of America; Helmholtz Centre for Infection Research, Germany

## Abstract

Effective treatment of infections caused by the bacterium *Staphylococcus aureus* remains a worldwide challenge, in part due to the constant emergence of new strains that are resistant to antibiotics. The serine/threonine kinase PknB is of particular relevance to the life cycle of *S. aureus* as it is involved in the regulation of purine biosynthesis, autolysis, and other central metabolic processes of the bacterium. We have determined the crystal structure of the kinase domain of PknB in complex with a non-hydrolyzable analog of the substrate ATP at 3.0 Å resolution. Although the purified PknB kinase is active in solution, it crystallized in an inactive, autoinhibited state. Comparison with other bacterial kinases provides insights into the determinants of catalysis, interactions of PknB with ligands, and the pathway of activation.

## Introduction

The gram-positive bacterium *Staphylococcus aureus* is a serious human pathogen that is responsible for an increasing number of illnesses and deaths each year [1]. The bacterium colonizes the nose and skin of humans and can cause illnesses ranging from skin infections [2] to life-threatening diseases such as endocarditis, bacteremia, pneumonia, meningitis, osteomyelitis, sepsis and the toxic shock syndrome [3], [4], [5], [6]. Successful treatment of *S. aureus* infections remains a challenge as drug-resistant strains, such as methicillin-resistant and vancomycin-resistant *S. aureus* (MRSA and VRSA, respectively), are gaining prominence. Furthermore, the emergence of community-acquired *S. aureus* strains forms a rapidly emerging public health problem [7]. In order to develop new strategies to combat these bacteria, a better understanding of the organisms and the functions of its components is needed.

**Figure 1:**
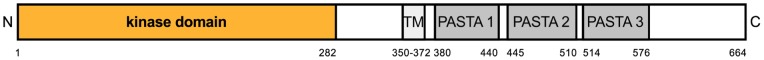
Domain structure of *S. aureus* PknB. The kinase region (PknB_SA-KD_) is shown in orange. TM: transmembrane domain, PASTA: penicillin-binding protein and serine/threonine kinase associated domains.

To overcome stressful conditions imposed by its host, *S. aureus* has developed various protective and offensive responses such as the sensing of environmental stimuli and the activation and inactivation of response regulators. This is generally achieved through cascades of phosphorylation reactions in the host, which in turn points to a key role of protein kinases in staphylococcal persistence. Protein kinases regulate a multitude of processes and signal transduction pathways in prokaryotes and eukaryotes [8]. A subgroup, the serine/threonine kinases (STKs), was originally thought to only be present in eukaryotic cells. However, in recent years STKs have also been identified in bacteria [9], [10], and these have been classified as eukaryotic-type serine/threonine kinases [11]. While many microorganisms encode for several eukaryotic-type STKs, *S. aureus* encodes only for one such protein, which has been termed PknB, PrkC or Stk1 by different research groups [11], [12], [13] and will be referred to as PknB here. PknB was originally identified through a transposon mutagenesis approach and is conserved in all *S. aureus* strains [14]. The kinase is composed of an N-terminal, cytosolic kinase domain, a central transmembrane domain, and three C-terminal, extracellular PASTA (penicillin-binding protein and serine/threonine kinase associated) domains (Fig. 1). PASTA domains are constructed from about 65–70 amino acids and are thought to bind beta-lactam compounds as well as peptidoglycans [15], [16]. The number of PASTA domains present in eukaryotic-type STKs can vary. *S. aureus* PknB and *B. subtilis* PrkC have both three PASTA domains, while the PknB of *M. tuberculosis* contains four such repeats [11], [12], [17].

PknB is of particular relevance for *S. aureus* survival and pathogenesis as it helps to regulate purine biosynthesis, autolysis, and other central metabolic processes of the bacterium and is involved in antibiotic resistance [12], [18], [19]. Moreover, recent data show that PknB can also act on human cellular proteins, and that these potential targets are involved in apoptosis, immune responses, transport, and metabolism [20]. The recently discovered secretion of PknB may also help the bacterium to evade intracellular killing and facilitate its growth [20]. Proper function of PknB is important for full expression of *S. aureus* pathogenesis, and it is also likely that phosphorylation levels controlled by PknB are essential in controlling bacterial survival within the host [21].

Structural information on *S. aureus* PknB is so far limited to the three PASTA domains that constitute the extracellular portion of the protein [11], [17]. Structural analyses of PknB homologs, such as PknB from *M. tuberculosis* [22], [23], [24], [25], [26] have provided insights into the overall fold of the cytosolic kinase domain and its interactions with ligands. However, it is well established that kinases adopt similar folds but differ in subtle ways in order to achieve their specificity. Here, we report a structural analysis of the kinase domain of *S. aureus* PknB in complex with a non-cleavable ATP analog, adenosine 5′-(β, γ-imido)-triphosphate (AMP-PNP). Comparison with other bacterial STKs provides insights into the determinants of PknB catalysis, its state of activation, and its interactions with potential ligands.

## Materials and Methods

### Cloning, expression and purification

DNA encoding the kinase domain of *S. aureus* PknB (PknB_SA-KD_) (residues 1–291) of *S. aureus* strain 8352 (GenBank accession number BAB42315) was amplified by PCR, and NdeI and XhoI cleavage sites and an additional stop codon were introduced. After digestion with NdeI and XhoI, the PCR product was inserted into the pET28b vector (Novagen), which includes an N-terminal His_6_-tag followed by a thrombin cleavage site, for protein expression in *E. coli* strain BL21-DE3. Transformed bacteria were grown in LB medium supplemented with 30 µg/ml kanamycin at 37°C to an optical density of 0.3 at 600 nm. After lowering the temperature to 20°C, the bacteria were induced by addition of 1 mM isopropyl-β-thiogalactopyranoside. After 24 hours of expression, bacteria were harvested by centrifugation and resuspended in 20 mM HEPES, 150 mM NaCl, 20 mM imidazole and 1 mM phenylmethanesulfonyl fluoride at pH 7.4. The sonified lysate was clarified by centrifugation and filtered. The solution was loaded onto a HisTrap column (GE Healthcare), which was then washed with lysis buffer. The His-tagged PknB_SA-KD_ was eluted with a linear imidazole gradient ranging from 10 to 500 mM. After reducing the imidazole concentration by alternating concentration and dilution steps, the protein was cleaved with thrombin (1 U/mg protein; GE Healthcare) for 24 h at 20°C. Cleavage was followed with a second Ni^2+^-affinity run to remove thrombin and uncleaved PknB_SA-KD_. The 33.1 kDa PknB_SA-KD_ was concentrated and purified by gel filtration (Superdex 75, GE Healthcare) in 20 mM HEPES, 150 mM NaCl at pH 7.4 (Fig. S1A). SDS-PAGE confirmed the purity of the product (Fig. S1B). Mass spectrometry analysis was used to verify the identity of PknB_SA-KD_, and circular dichroism spectroscopy (CD-spectroscopy, Jasco J -720) confirmed that it was folded (Fig. S1C). PknB_SA-KD_ was used at a concentration of 0.2 mg/mL in 2.5 mM HEPES, 18.75 mM NaCl at pH 7.4 for CD-spectroscopy measurements at room temperature. The path length was 0.1 cm, and data were acquired at a scanning speed 100 nm/min with a data pitch of 0.5 nm.

### Chemical cross-linking

Purified PknB_SA-KD_ was incubated with different concentrations of glutaraldehyde for 15 min at room temperature. The reaction was stopped by adding 4xSDS-protein buffer and incubating the mixture at 95°C for 5 minutes. Identical procedures were carried out with the 22 kDa adenovirus type 21 fiber knob (Ad21), which forms trimers and served as a positive control, and with the 25kDa chymotrypsinogen A (Sigma-Aldrich), which is monomeric and served as the negative control.

### Protein kinase assay

In vitro phosphorylation of 25 µg/mL PknB_SA-KD_ was performed for the indicated time at 37°C with 1 µg/mL myelin basic protein (MBP, Sigma, Deisenhofen, Germany) and 4 µCi γ^33^-ATP/µl in 20 µl reaction buffer containing 50 mM HEPES (pH 7.5), 1 mM DTT, 0.01% Brij35, 3 mM MnCl_2_ and 3 mM MgCl_2_. In each case, the reaction was stopped by adding 4xSDS-protein buffer. One-dimensional gel electrophoresis was performed and finally, radioactive proteins were visualized by autoradiography using direct-exposure film.

### Protein crystallization and structure determination

The final solution used for crystallization contained 8 mg/mL PknB_SA-KD_, 5 mM MgCl_2_, 4 mM AMP-PNP (tetralithium salt hydrate), 2% (w/v) benzamidine hydrochloride and 1 mM dithiothreitol in 20 mM HEPES, 150 mM NaCl at pH 7.4. Crystals were grown with the sitting drop vapor diffusion method by mixing equal amounts of protein solution and crystallization solution (80 mM 2-(N-morpholino)ethanesulfonic acid pH 6.0, 1.3 M sodium citrate (pH 7.0), 2% (w/v) benzamidine hydrochloride and 60 mM MgCl_2_ at 4°C. Crystals appeared after several days and grew to a maximum size of 150 µm diameter. Crystallization trials using mixtures of MnCl_2_ and MgCl_2_ or MnCl_2_ could not improve the crystal quality. They belong to space group C2 and contain six kinase domains in their asymmetric unit. The crystals were mounted on a loop and flash frozen in liquid nitrogen prior to data collection.

X-ray diffraction experiments were performed at the X06SA beam line of the Swiss Light Source, Paul Scherrer Institut, Villigen, Switzerland. Data extending to 3.0 Å resolution were recorded using the PILATUS detector and processed with XDS [27]. Initial phases were determined with PHASER [28] using the *M. tuberculosis* PknB structure as search model (Protein data bank (PDB) ID: 1O6Y [23]). The search model was modified by truncating side chains that differed in sequence from the *S. aureus* protein, and by removing loops and bound ligands. Molecular replacement yielded one solution containing six copies that gave rise to a sensible crystal packing. The initial model was then improved through alternating steps of model building in Coot [29] and refinement in Phenix [30]. The refinement parameters included simulated annealing, Ramachandran refinement, and non-crystallographic symmetry (NCS) restraints. Eight groups per chain were defined in the NCS refinement, excluding the most flexible loops. The fragments of chains A, B and C, which were similar to each other, were defined as NCS-linked groups, and the same was done for chains D, E and F. Electron density for AMP-PNP appeared in all six chains during the course of the refinement, allowing the incorporation of the ligand into the model. The final structure has good quality, with R_work_ and R_free_ values [31] of 21.49 and 24.64%, respectively. Geometric restraints for the AMP-PNP ligand were calculated using the PRODRG server [32]. Data and refinement statistics are given in Table 1. Atomic coordinates und structure factors have been deposited in the PDB (http://www.pdb.org) under the accession code 4EQM.

**Table 1:** Data collection and refinement statistics.

Parameter	Value
**Data collection**	
Beam line	X06SA, SLS
Wavelength (Å)	1.0000
Space group	C2
Cell dimensions	
* a, b, c* (Å)	221.51, 127.55, 70.28
α, β, γ (°)	90.00, 89.96, 90.00
Resolution (Å)	45.0–3.0 (3.08–3.00)α
* R*_meas_	4.8 (46.0)α
I/σI	17.99 (2.68)α
Completeness (%)	99.1 (99.5)α
Unique reflections	38,868 (2,889)α
Redundancy	3.39 (3.38)α
Wilson B (Å^2^)	95.43
**Refinement**	
Resolution (Å)	43.5–3.0
* R*_work_/*R*_free_	0.2149/0.2464 (0.3403/0.3962)α
No. of atoms	12,609
Protein	12,420
Ligands	189
* B*-factors (Å^2^)	
Protein	97.1
Benzamidine/AMP-PNP	87.9/105.1
r.m.s.β deviations	
Bond lengths (Å)	0.005
Bond angles (°)	0.918
Ramachandran plot	
Most favored regions (%)	95.7
Allowed regions (%)	4.3
Disallowed regions (%)	0.0

α Values in parentheses are for highest resolution shell.

β r.m.s., root mean square.

## Results

### Activity assay

**Figure 2:**
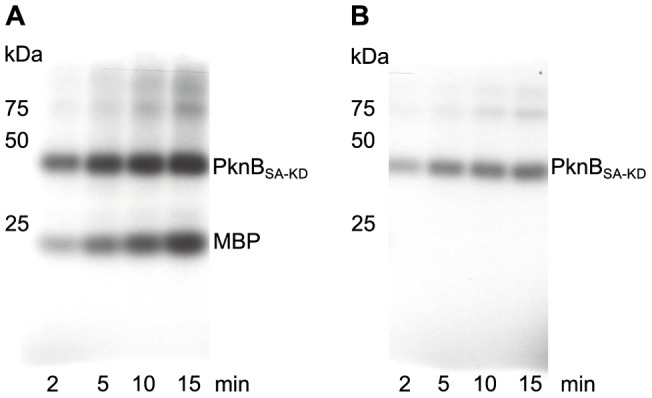
Activity test of PknB_SA-KD_. PknB_SA-KD_ (25 ng) was incubated either with myelin basic protein (MBP; 1 µg) (A) or alone (B) together with γ^33^-ATP, MnCl_2_ and MgCl_2_ for the time indicated. Position and size (kDa) of molecular weight markers are indicated on the left side. Phosphorylation of MBP (A) and autophosphorylation (B) are visualized by autoradiography using direct-exposure film. The phosphorylation rate is increasing as a function of time in both experiments, demonstrating that the purified PknB_SA-KD_ protein is active.

The functional activity of the kinase domain of *S. aureus* PknB (PknB_SA-KD_) was tested by an *in vitro* phosphorylation assay. PknB_SA-KD_ is able to phosphorylate other proteins such as myelin basic protein (MBP) in an efficient manner (Fig. 2A). The target protein MBP was previously used as a surrogate substrate for activity tests of the full-length PknB [12] and of *Mycobacterium tuberculosis* PknB [33]. Additionally, PknB_SA-KD_ is able to perform autophosphorylation (Fig. 2B).

### Overall Structure

**Figure 3:**
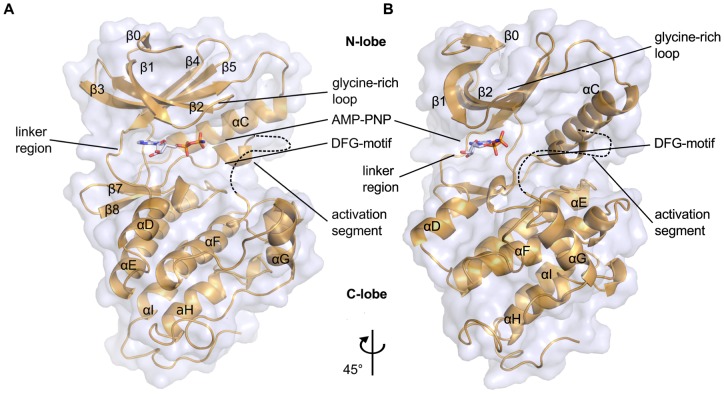
Overall structure of PknB_SA-KD_ in complex with AMP-PNP. The two views differ by a rotation of 45° around a vertical axis. The AMP-PNP ligand is located in the cleft between the N- and the C-lobe. Due to its high flexibility, the terminal phosphate group of AMP-PNP is not visible in the electron density and is therefore not shown here.

**Figure 4:**
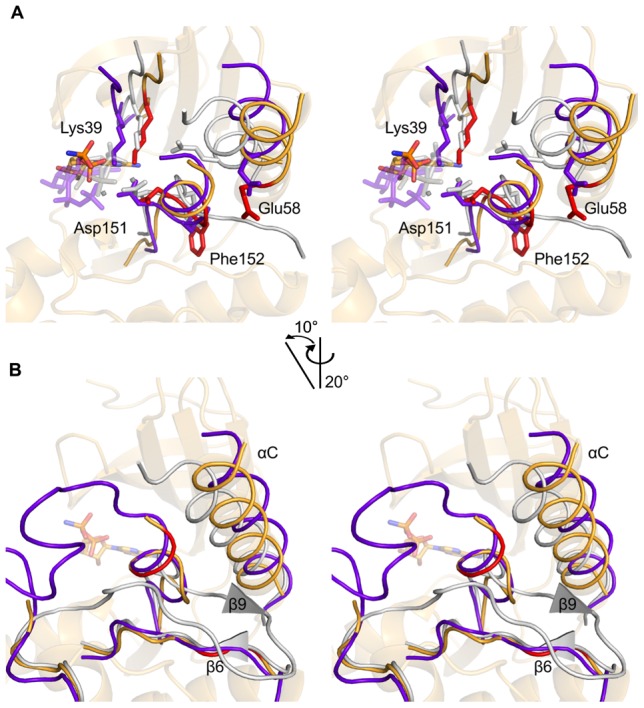
Stereo views of the activation site of PknB_SA-KD_. (A). The PknB structure is shown in light orange. It was superimposed onto the kinase structures of active PKA (grey, PDB ID: 1ATP [35]) and inactive c-Src (purple, PDB ID: 2SRC [36]) using C-lobe residues 100–250. The highly conserved residues Lys39, Glu58, Asp151 and Phe152 of PknB_SA-KD_ are highlighted as red sticks. The latter two residues are part of the DFG-motif. Corresponding residues Lys72, Glu91, Asp184 and Phe185 of PKA, as well as the backbone of PKA and ATP, are colored in grey. Corresponding residues Lys295, Glu310, Asp404 and Phe405 of c-Src, as well as the backbone of c-Src and ATP, are colored in purple. Mn^2+^ ions in the PKA structure are shown as small gray spheres. (B). Close-up view of the β-sheet formed by β6 and β9 in active kinases such as PKA. The colors are the same as in A. The β-sheet in PKA is represented with triangles as the β-strands only consist of two residues each. The red part of PknB_SA-KD_ represents residues Ile129, Val130, Lys156 and Ala157, which are the residues that would form strands β6 and β9 in the active protein.

PknB_SA-KD_ exhibits the typical kinase fold, with N- and C-lobes creating a central ligand binding region that serves to accommodate the substrate or its analog AMP-PNP (Fig. 3). The N-lobe comprises residues 1 to 90 and contains a six-stranded, antiparallel β-sheet (strands β0-β5) that packs against the αC-helix. The C-lobe is composed of six α-helices and a small two-stranded β-sheet (strands β7-β8). Many kinases contain two additional strands, β6 and β9, which form a second β-sheet in the C-lobe. This small sheet is absent in PknB_SA-KD_ due to the conformation of the activation segment (Fig. 4). The two lobes in PknB_SA-KD_ are connected *via* the linker region (residues 87–92) and *via* a loop that leads from the C-terminus of the αC-helix to the β4 strand (Fig. 3A, B). Although present in the crystallized PknB_SA-KD_, residues 284 to 291 at the C-terminus are not well defined by electron density and could not be built. We conclude that the kinase domain of *S. aureus* PknB includes residues 1–282 (Fig. 1), in contrast to the computer aided residue assignment for the kinase region in Donat et. al, 2009 [12] (residues 10–268) and the longer PknB_SA-KD_ sequence used for crystallization. As is the case in many kinase structures, high flexibility of the activation loop (residues 160–171) results in this segment not being traceable in the electron density maps. The six PknB_SA-KD_ structures present in the asymmetric unit (molecules A to F, respectively) differ in several surface-exposed loops as a result of non-identical crystal contacts. The molecules can be divided into two homogeneous groups; molecules A, B and C bind benzamidine in a similar location and form similar crystal contacts. However, these features are not conserved in molecules D, E and F. The electron density for molecules A, B and C is more detailed in most regions, allowing unambiguous assignment of most side chains orientations. By contrast, the electron density for chains D, E and F is less well defined, and the density for the ATP-binding site is also somewhat different in these three chains. The β-phosphates of AMP-PNP are arranged in a different orientation in chains D-F compared with chains A-C. The main chain B-factor plot (Fig. S2) shows overall agreement of the B-factor distribution in all six chains. Residues forming a secondary structure element have significantly lower B-factors compared to residues in flexible loop regions. This flexibility is also reflected in the high overall B-factor. The B-factor differences between the chains A, B and C are small, the same is true for chains D, E and F. It is likely that variation in PknB_SA-KD_ phosphorylation contribute to the observed differences in electron density. Unless specified otherwise, molecule A will be used to discuss the salient features of PknB_SA-KD_.

### AMP-PNP binding

The ATP analog AMP-PNP is bound in the cleft separating the two lobes of the kinase (Fig. 3). The adenine ring projects deep inside the cleft, into a pocket that is largely hydrophobic. Two hydrogen bonds, contributed by the backbone amide of Ile90 and the backbone oxygen of Glu88, anchor the adenine ring (Fig. S3). The ribose and phosphate moieties of AMP-PNP do not make equivalent contacts in the different chains, and these contacts are therefore probably not significant. Furthermore, the γ-phosphate group is not visible in the electron density maps, suggesting that the lack of contacts with PknB_SA-KD_ increases its flexibility.

### PknB_SA-KD_ is in an inactive conformation

**Figure 5:**
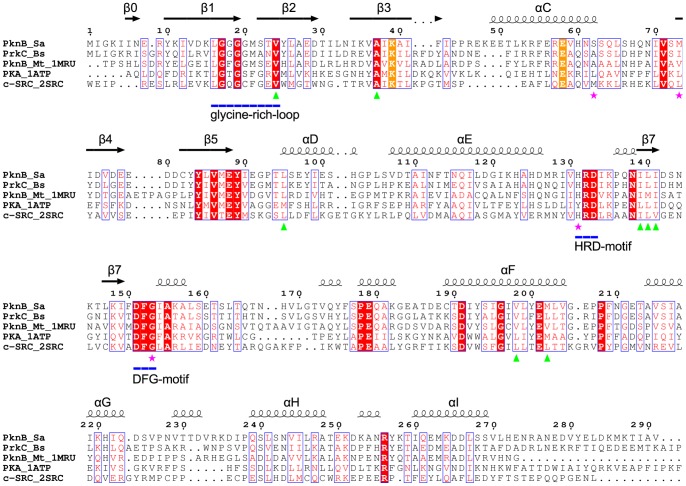
Structure-based sequence alignment. Structure-based sequence alignment of the *S. aureus* PknB kinase domain with the kinase domains of *B. subtilis* PrkC (no structure available), *M. tuberculosis* PknB (PDB ID: 1MRU [24]), murine cAMP dependent Protein Kinase A (PDB ID: 1ATP [35]; PDB ID: 1CTP [34]) and human tyrosine protein kinase c-Src (PDB ID: 2SRC [36]). The secondary structure of PknB_SA-KD_ is shown above the alignment and the numbering of the sequences corresponds to *S. aureus* as well. The HRD- and DFG-motifs and the glycine-rich loop are underlined in blue. The highly conserved residues Lys39 and Glu58 are marked in orange. Green triangles indicate the residues of the C-spine; magenta stars mark residues of the R-spine.

In order to determine whether the structure of PknB_SA-KD_ is in an active or an inactive conformation, we have compared it with the following kinase structures: (i) PknB of *M. tuberculosis*, which is the first reported structure of a bacterial STK (PDB ID: 1O6Y [23] and 1MRU [24]; (ii) cAMP-dependent protein kinase A (PKA) in an open conformation (PDB ID: 1CTP [34]); (iii) a closed, active PKA structure with bound ATP (PDB ID: 1ATP [35]); and (iv) human c-Src, a tyrosine kinase in the autoinhibited conformation (PDB ID: 2SRC [36]). A structure-based sequence alignment was generated for all five kinases using ClustalW [37], Espript [38], Strap [39] (Fig. 5), and superpositions were performed by aligning residues 100–250 of the C-lobe of PknB_SA-KD_ with the other structures. Although there is no crystal structure available for it, we also included the sequence of the *B. subtilis* protein kinase C (PrkC) in our analysis because of its especially high homology to the *S. aureus* PknB sequence and the fact that both kinases have three extracellular PASTA domains.

**Figure 6:**
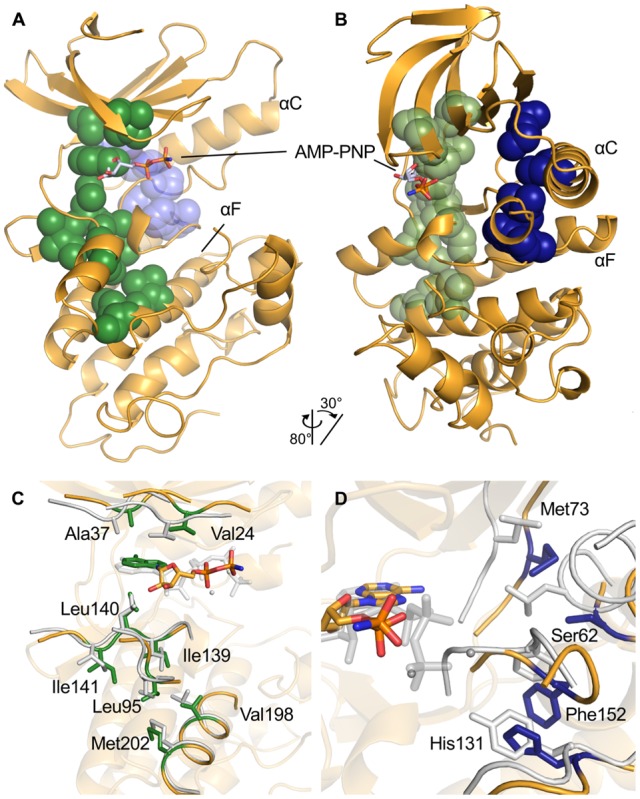
The C- and R-spine regions of PknB_SA-KD_. (A, B). Overview of the location of the two spines in PknB_SA-KD_. The C-spine is colored in green, the R-spine in the background in blue. The two views differ by the indicated rotation to provide a better view of the orientation and location of the R-spine. (C). Detailed view of the residues belonging to the C-spine of PknB_SA-KD_ and the adenine of AMP-PNP as part of the spine are shown in green. The residues of the C-spine of PKA in a closed state (PDB ID: 1ATP [35]) are shown for comparison. (D). Detailed view of the R-spine residues of PknB_SA-KD_ in blue. Corresponding residues of PKA are shown in grey. While the spine is formed in PKA, it is interrupted by the Ser62 and placed away from ideal position in PknB_SA-KD_. The structure of PKA in panels C and D was aligned with PknB_SA-KD_ C-lobe residues 100–250.

Our analysis highlights several highly conserved residues that are important for kinase function: the glycine-rich loop, the DFG-motif, the position of the αC-helix, and the catalytic and regulatory spines (C-spine and R-spine, respectively). Representative F_obs_-F_calc_ omit electron density maps of the ATP binding regions in chains A and D are shown in Figs. S3A and S3B, respectively. F_obs_-F_calc_ omit electron density maps of the DFG-motif and the inhibition helix of chain A, the AMP-PNP and its surroundings, and the αF-helix of chains A and D are shown in Fig. S4A–D. The glycine-rich loop positions the γ-phosphate of ATP in an orientation that facilitates phosphoryl transfer during catalysis [40]. The DFG-motif is near the activation segment (Fig. 3). The C- and R-spines are sets of non-contiguous hydrophobic residues that line the interior of a kinase and stabilize its active, closed conformation (Fig. 6A, B) [40], [41], [42], [43].

#### a. Stabilization of the phosphates

Connecting strands β1 and β2 in the N-lobe, the glycine-rich loop has a consensus sequence of GX_1_GX_2_φGX_3_V, with φ denoting a hydrophobic residue (usually tyrosine or phenylalanine) and X indicating any amino acid [23], [44], [45]. Since it serves to stabilize the phosphates of ATP, this loop is also sometimes referred to as phosphate binding loop or P-loop [45]. In the active state of kinases, the glycine-rich loop faces the terminal phosphates of ATP, positioning the γ-phosphate for the transfer reaction [46], [47], [48]. In contrast, the glycine-rich loop faces away from ATP in open and inactive kinases such as PKA (in PDB ID: 1CTP [34]) (Figs. S3, S5). While the glycine-rich loop is strictly conserved in PKA, c-Src, and many other kinases, its consensus sequence is altered to GX_1_GGMX_2_X_3_V in PknB_SA-KD_ and the two other bacterial kinases included in our comparison. Moreover, the X_1_ residue is also a glycine in both *B. subtilis* PrkC and PknB_SA-KD_. The increased number of directly linked glycines would likely render the loop more flexible in both proteins. The comparison of the structures shows that the glycine-rich loop in PknB_SA-KD_ is in a conformation that differs from those observed in both active and inactive kinases (see above) (Figs. S3, S5). Its “tip” (residues 18 to 20) is twisted upwards, facing away from the phosphates. However, its “base” (residues 22–25) is still in a position similar to those observed in active kinases.

In active kinases, the phosphates of ATP are stabilized in the cleft between the two lobes. The motif involved in binding phosphates on the C-lobe side is the DFG-motif. Protein kinases stabilize the phosphates of bound ATP with magnesium ions, which in turn are ligated to an aspartic acid in the DFG-motif (residues 151–153 in PknB_SA-KD_). In active kinases, the DFG-motif also features an internal hydrogen bond between the aspartic acid and the glycine, and a second hydrogen bond between the glycine and the amide nitrogen of residue DFG+2 (Ala155 in PknB_SA-KD_) [41]. The DFG-motif of PknB_SA-KD_ lacks internal hydrogen bonds, and no magnesium ion is visible in the vicinity of Asp151. It is clearly not in an active conformation and does not stabilize the phosphate groups of AMP-PNP.

A salt bridge is located close to the DFG-motif in active kinases. To enable an active kinase conformation, the αC-helix must be oriented such that a salt bridge between the strictly conserved residues Glu58 and Lys39 can be formed. Lys39 lies in the β3-strand of the N-lobe and helps to stabilize the α- and β-phosphates of ATP [46], [47], [48]. Structural comparison with other active kinases shows that the αC-helix of PknB_SA-KD_ is not in a closed conformation (Fig. 4 and Fig. S4B). The helix is rotated away from the active site, and Glu58 does not form a salt bridge with Lys39.

#### b. The C- and R-spines

These spines stabilize the active, closed conformation of a kinase and contain residues from both lobes of the kinase. The C-spine attaches the active site to the αF-helix, connecting the two lobes *via* the adenine ring system. It lines the rear of the adenosine-binding pocket and stabilizes the closed, active conformation of protein kinases. In PknB_SA-KD_, the C-spine is formed by residues Val24, Ala37 in the N-lobe and residues Ile139, Leu140, Ile141, Leu95, Val198 and Met202 in the C-lobe (Fig. 6A, C). The C-spine of PknB_SA-KD_ and the bound adenine ring superimpose well with those of the closed, active PKA structure (Fig. 6C).

The R-spine also serves to stabilize the active conformation of protein kinases [40], [41], [42], [43]. In PknB_SA-KD_, the putative residues for the spine are Met73, Ser62, His131 and Phe152. The latter residue is part of the DFG-motif. Asp191 is stabilizing the backbone of His131, thereby anchoring the spine to the αF-helix. Ser62 lies in the αC-helix and is located four residues C-terminal to the highly conserved Glu58. Since the DFG-motif and the αC-helix are not in an active conformation, the R-spine cannot be fully formed in PknB_SA-KD_ (Fig. 6B, D).

#### c. Inhibition helix

In order to assume an active state, the αC-helix of PknB_SA-KD_ would have to change its position (Fig. 4). This is however not possible in our structure because the space into which the αC-helix would have to rotate is already occupied by the activation segment located directly after the DFG-motif (Fig. 4). In active kinases the activation loop makes close contacts to the C-lobe [40] and forms the β-sheet between strands β6 and β9 (Fig. 4B). The activation segment of PknB_SA-KD_ interacts with the N-lobe and the αC-helix. The activation segment forms a short helix directly after the DFG-motif and blocks the area for the αC-helix to assume an active conformation. The putative residues of β9 are part of the inhibition helix and far away from β6, so that the β-sheet cannot be formed. The absence of the β6/β9 sheet is also a marker for an inactive conformation of PknB_SA-KD_ (Fig. 4B). The PknB_SA-KD_ αC-helix is stabilized by a hydrophobic interface similar to the one found in the structures of CDK2 and c-Src in their autoinhibited conformations [36], [49], [50]. The interface is formed by several residues in the αC-helix, strands β3 and β4, and the activation segment.

### Analysis of surface conservation

**Figure 7:**
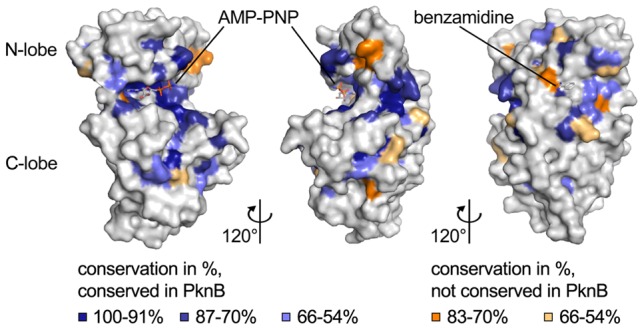
Analysis of conservation of PknB residues. Surface representation of PknB_SA-KD._ The three views differ by rotations of 120° and 240°, respectively, around a vertical axis. The coloring is based on an alignment of 24 bacterial STKs (Fig. S6). Blue indicates highly conserved residues (100–91% conservation in dark blue, 87–70% conservation in blue, and 66–54% light blue). Residues that are highly conserved in most kinases but are different in PknB_SA-KD_ are colored in orange (83–70% conservation in orange, 66–54% conservation in light orange). In the right panel, the benzamidine bound to PknB_SA-KD_ in three of the six chains of the asymmetric unit is shown as a stick model. The benzamidine is not visible in the other two panels.

In order to identify conserved features and compare them with homologous proteins, the PknB_SA-KD_ sequence was aligned with representatives of several bacterial STKs (Fig. S6). For our analysis, we selected the bacterial STKs recently analyzed by Pereira *et al*. [10]. All analyzed kinases exhibit strong conservation in the prototypical regions required for catalytic function, such as the DFG-, HRD- and SPE-motifs and the glycine-rich loop. In order to depict the location and distribution of conserved residues, we mapped them onto the surface of PknB_SA-KD_ (Fig. 7). As expected, residues important for the catalytic function of the kinase are highly conserved (highlighted blue in Fig. 7). These residues cluster in the ATP-binding site, the glycine-rich loop, and the DFG-, HRD- and SPE-motifs. However, a small number of residues are highly conserved in most other kinases but differ from the consensus sequence in PknB (highlighted orange in Fig. 7). The remaining surface of PknB_SA-KD_ is remarkably devoid of conserved residues.

## Discussion

We report here the crystal structure of the catalytically competent kinase region of *S. aureus* PknB. In its physiological context, the kinase is attached to the bacterial cell wall *via* a membrane anchor, and it phosphorylates substrates in response to stimuli that engage the extracellular PASTA domains. Activity assays demonstrate that purified PknB_SA-KD_ is able to phosphorylate substrates efficiently. However, an analysis of structural parameters that define the active states of protein kinases clearly demonstrates that PknB_SA-KD_ has crystallized in an inactive conformation. Although it does bind the ATP analog AMP-PNP, this substrate is not bound in a conformation that would enable catalysis. The AMP-PNP triphosphate moiety is not contacted by either a magnesium ion or residues from the glycine-rich loop or the DFG-motif. As PknB_SA-KD_ is catalytically active in solution, it is conceivable that it exists in different conformations, corresponding to active and inactive states, in solution, perhaps owing to different states of phosphorylation. Hence, crystallization likely selected the inactive state.

### Dimer formation

**Figure 8:**
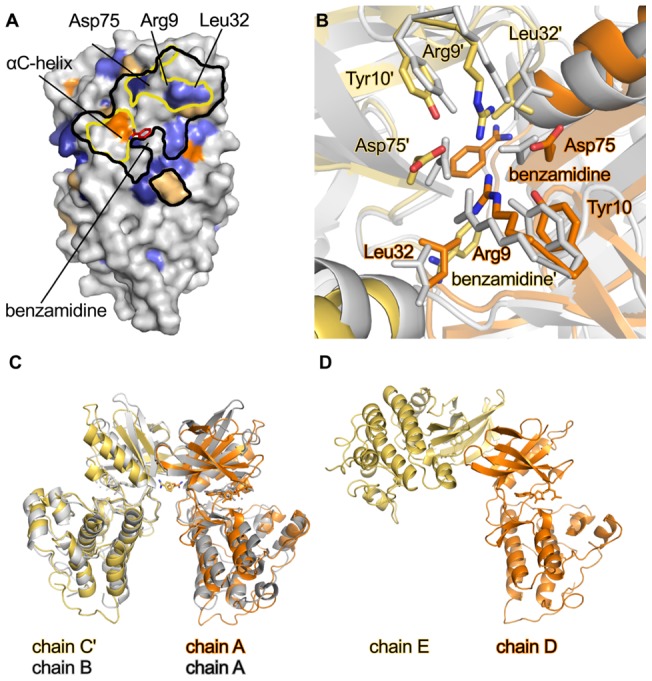
Analysis of PknB_SA-KD_ crystal contacts. (A). Footprint of contacts between a symmetry mate of molecule C (C′) and molecule A in the crystals. Molecule A is shown in surface representation. Areas within black lines indicate crystal contacts with molecule C′ (distance <4.5 Å). Areas within yellow lines indicate the residues involved in dimer formation [22]. The surface conservation is shown as in Fig. 7, and the benzamidine is bound to chain A of PknB_SA-KD_ is shown as a red stick model. Molecule pairs B/B′ and C′/A form similar crystal contacts. (B). Detailed view into the dimer interface formed by the A/C′ dimer. Chains A and C′ of PknB_SA-KD_ are shown in orange and yellow, respectively. Chains A and B of dimeric *M. tuberculosis* PknB (PDB ID: 1MRU [24]) are in grey. Residues Arg9, Tyr10, Leu32 and Asp75, which are involved in dimer formation, are represented with sticks. These residues were also used for superimposing the two dimers. (C). Overview of the orientation of the dimer of *M. tuberculosis* PknB and PknB_SA-KD_. The colors are the same as in panel B. The N-lobes of PknB_SA-KD_ chain A and *M. tuberculosis* PknB chain A were superposed. (D). Crystal contact involving chains D and E in PknB_SA-KD_. The orientation of chain D is the same as that of chain A in panel C.

An attractive scenario for PknB activation could be based on dimer formation, and dimerization has in fact been implicated in the regulation of the activity of the *M. tuberculosis* PknB kinase domain. In that case, the kinase domain forms dimers that are stabilized by a salt bridge between Arg9 in one monomer and Asp75 of another monomer [22], [51]. Although PknB_SA-KD_ consistently eluted as a monomer in gel filtration experiments in solution (Fig. S1A), it is conceivable that low-affinity dimerization of PknB_SA-KD_ could occur at higher concentration. This hypothesis was evaluated by chemical crosslinking experiments using glutaraldehyde (Fig. S7). No crosslinked dimer of PknB_SA-KD_ was obtained under any of the tested conditions, while a control protein known to form trimers could be successfully cross-linked under identical conditions. Additionally, inspection of the crystal packing can sometimes provide clues about the possible existence of oligomers. We find that three of the six PknB_SA-KD_ molecules present in the crystals form nearly identical dimers with their symmetry mates (A–C′, B–B′, C–A′), and the arrangement of these putative dimers resembles the *M. tuberculosis* PknB dimer (Fig. 8). It is therefore conceivable that activation of *S. aureus* PknB also involves dimerization. We note, however, that the putative PknB_SA-KD_ dimer interface contains benzamidine, a compound that was present in the crystallization solution and that was required to obtain good-quality crystals. The observed dimer may therefore be a crystallization artifact. Moreover, the three remaining molecules in the crystals (chains D, E, F) do not form similar dimers.

It is of course possible that dimerization of the kinase region is linked to its phosphorylation status, and that the copies of PknB_SA-KD_ present in the crystals have different states of phosphorylation, thus impacting their dimerization properties.

Another, perhaps more likely, scenario could link dimerization to the extracellular PASTA domains, which could alter their association state in response to signal binding [11], [17].

### Inhibition helix

Although it represents an inactive conformation of the kinase, the PknB_SA-KD_ crystal structure nevertheless provides insights into a pathway of activation. In order to activate PknB_SA-KD,_ the αC-helix would need to significantly change its location by rotating into the binding site. Such a rotation, however, is not possible in our crystal structure because the space that would accommodate the rotated αC-helix is already occupied by the activation segment, which forms a short helix directly following the DFG-motif (Fig. 4 and Fig. S4A). The activation loop of active kinases makes close contacts with the C-lobe, whereas in PknB_SA-KD_ it interacts with the N-lobe and the αC-helix.

The activation segment would have to be displaced to allow for the formation of an active PknB conformation. This could be triggered by autophosphorylation in the activation segment. Thr164 could play a key role in this conformational change. Structural alignments with inactive c-Src kinase reveal a similar orientation of the activation segment, with an autoinhibition helix blocking the inward rotation of the αC-helix [36], [52] (Fig. 4). In this case, c-Src can be activated through the phosphorylation of residue Tyr416, which is the residue equivalent to Thr164 in PknB.

A dissolving of the inactive state of the activation segment could conceivably trigger a downward movement of the glycine-rich loop to stabilize the β- and γ-phosphates of the ATP. Due to weak electron density as a result of flexibility, it is not possible to model the activation segment. However, difference electron density in this region suggests that parts of the activation loop lie next to the glycine-rich loop, preventing interaction of the glycine-rich loop with phosphates.

In conclusion, our structural study provides improved understanding of the function of eukaryotic like serine/threonine kinases in bacteria at the molecular level. Future work will aim at the identification of substrates of PknB, the molecular mechanisms of substrate selection and the role of autophosphorylation for the activity of the kinase. In particular, the role of ligand-dependent dimerization of extracellular PASTA domains for activation of PknB remains to be clarified. Moreover, recent work provided evidence that PknB is embedded in the tight regulatory network controlling virulence of *S. aureus*. The structural information presented in this study may serve as a basis for further investigations of the molecular mechanisms determining pathogenesis of the major human pathogen *S. aureus*.

## Supporting Information

Figure S1 **Biochemical and biophysical analysis of purified PknB**_SA-KD_. A. Size-exclusion chromatography run on Superdex 75 (PC 3.2/30). The elution profile of PknB_SA-KD_ is shown in red in comparison to standard proteins. B. SDS-PAGE of purified PknB_SA-KD_. C. CD-spectrum of purified PknB_SA-KD_.
(TIF)

Figure S2 **B-factor plot for the six chains of PknB_SA-KD_ present in the asymmetric unit.** Secondary structure elements are aligned below the plot, with α-helices colored in blue and β-strands colored in orange. The red line indicates the overall B-factor average for all six chains.
(TIF)

Figure S3 **Stereo view into the AMP-PNP binding site.** The depicted map is an omit map (F_obs_-F_calc_) of the ligand, contoured at 3.0 σ and drawn with a radius of 5Å around AMP-PNP. Panels A and B show omit maps of the AMP-PNP bound to chains A and D, respectively.
(TIF)

Figure S4 **Stereo views of representative omit maps of PknB_SA-KD_.** All panels show F_obs_-F_calc_ omit electron density maps contoured at 1.0 σ. In panel A the omit map for the DFG-motif and the inhibition helix is shown. Panel B shows the phosphate binding region. Panels C and D show the omit map for the αF-helix for chain A in panel C and chain D in panel D. Maps were drawn with radii of 3Å (panels A, C and D) and 8Å (panel B) around the depicted coordinates. The larger radius for panel B was chosen to show that no extra density that would account for a magnesium ion exists in the vicinity of the AMP-PNP ligand.
(TIF)

Figure S5 **Orientation of the glycine-rich loop.** All kinases were aligned with the C-lobe of PknB_SA-KD_ (residues 100–250). The PknB_SA-KD_ structure is drawn in orange. The closed PKA structure is shown in green (PDB ID: 1ATP [35]), and the open PKA structure is shown in red (PDB ID: 1CTP [34]). The kinase domain of c-Src (PDB ID: 2SRC [36]) is shown in purple.
(TIF)

Figure S6 **Alignment of selected kinases and analysis of conservation**. The kinases were selected according to [10]. Five kinases were omitted due to lack of DFG-, SPE-, HRD-motif or the N-lobe. The color code is identical to that used in Fig 7. Blue indicates highly conserved residues (100–91% conservation in dark blue 88–74% in blue and 69–54% light blue). Residues highly conserved but different in PknB_SA-KD_ are colored in orange (88–71% in orange, 69–54% in light orange). The selected kinases are (Uniprot-ID in parentheses): *Staphylococcus aureus*, PknB (Q7A5Z8); *Bacillus subtilis*, PrkC (O34507); *Corynebacterium glutamicum*, PknA (Q8NU97); *Corynebacterium glutamicum*, PknB (Q8NU98); *Corynebacterium glutamicum*, PknG (Q6M299); *Corynebacterium glutamicum*, PknL (Q6M3Q8); *Mycobacterium tuberculosis*, PknA (P65726); *Mycobacterium tuberculosis*, PknB (P0A5S4); *Mycobacterium tuberculosis*, PknD (O05871); *Mycobacterium tuberculosis*, PknE (P72003); *Mycobacterium tuberculosis*, PknF (P72003); *Mycobacterium tuberculosis*, PknG (P65728); *Mycobacterium tuberculosis*, PknH (Q11053); *Mycobacterium tuberculosis*, PknI (P65730); *Mycobacterium tuberculosis*, PknJ (P65732); *Mycobacterium tuberculosis*, PknK (P95078); *Mycobacterium tuberculosis*, PknL (O53510); *Myxococcus xanthus*, Pkn4 (Q95478); *Myxococcus xanthus*, Pkn8 (Q9XBP6); *Myxococcus xanthus*, Pkn14 (Q93NE3); *Pseudomonas aeruginosa*, PpkA (Q9I758); *Streptococcus pneumonia*, StkP (Q8KY50).
(TIF)

Figure S7 **Chemical cross-linking of PknB_SA-KD_ and controls with glutaraldehyde.** Shown is an SDS-PAGE analysis of the crosslinking experiment. Untreated (ut) protein was loaded on the gel next to each protein as controls. The small bands in PknB_SA-KD_ lanes indicate a weak impurity of PknB_SA-KD_ at 80 kDa.
(TIF)
