# Supplementary figures and images for: Structural Analysis of Staphylococcus aureus Serine/Threonine Kinase PknB
Source: PLoS One. 2012 Jun 11;7(6):e39136. doi: 10.1371/journal.pone.0039136 (PMC3372466; doi:10.1371/journal.pone.0039136)

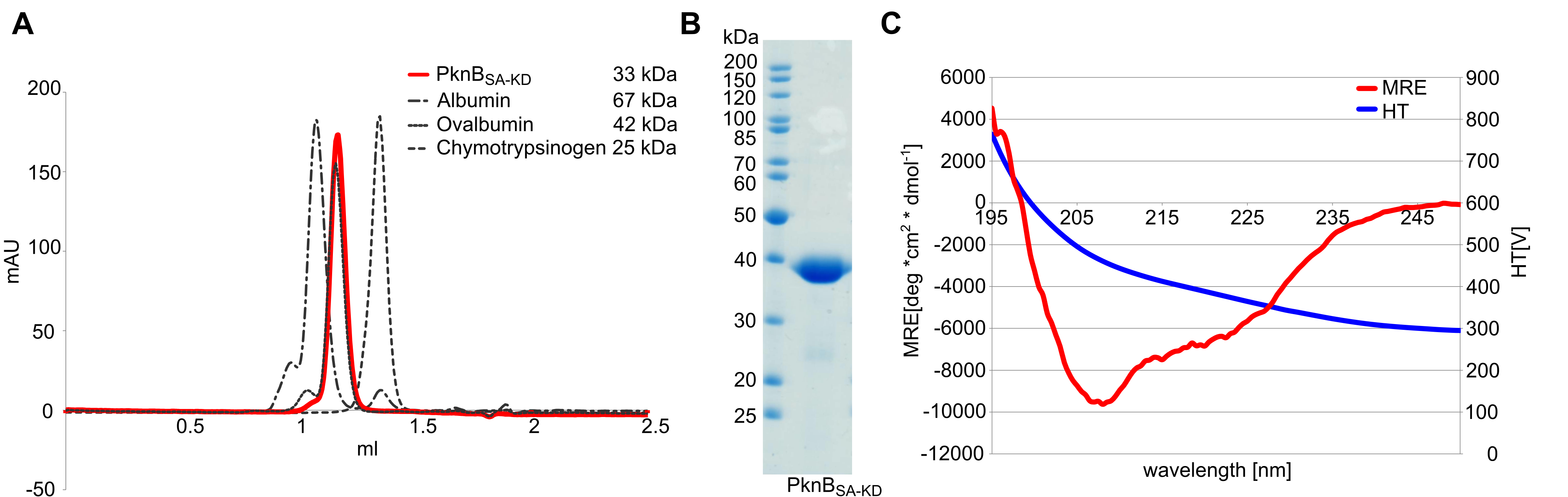

Supplement: Figure S1 — Biochemical and biophysical analysis of purified PknB SA-KD. A. Size-exclusion chromatography run on Superdex 75 (PC 3.2/30). The elution profile of PknBSA-KD is shown in red in comparison to standard proteins. B. SDS-PAGE of purified PknBSA-KD. C. CD-spectrum of purified PknBSA-KD. (TIF) [file pone.0039136.s001.tif]

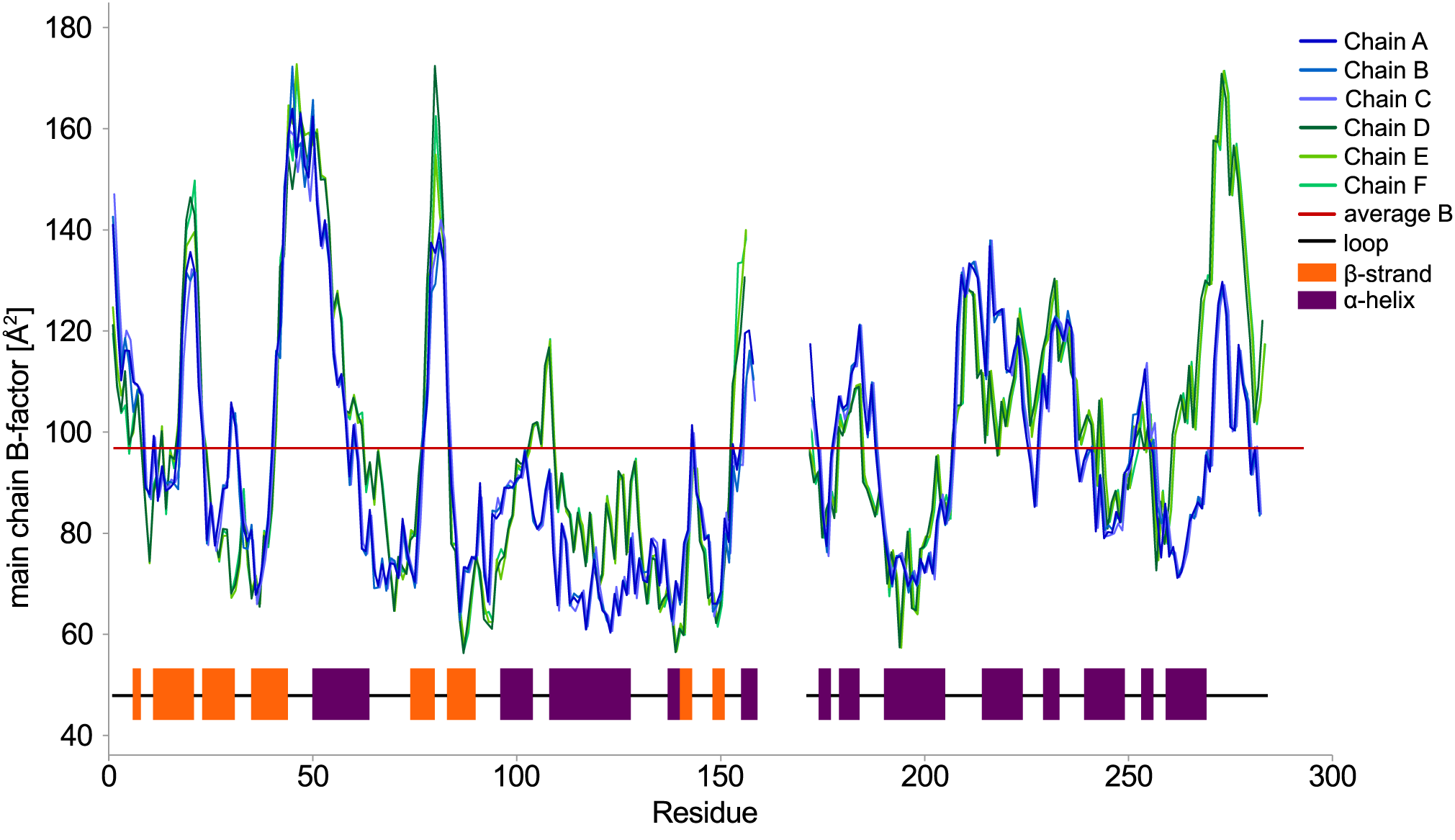

Supplement: Figure S2 — B-factor plot for the six chains of PknBSA-KD present in the asymmetric unit. Secondary structure elements are aligned below the plot, with α-helices colored in blue and β-strands colored in orange. The red line indicates the overall B-factor average for all six chains. (TIF) [file pone.0039136.s002.tif]

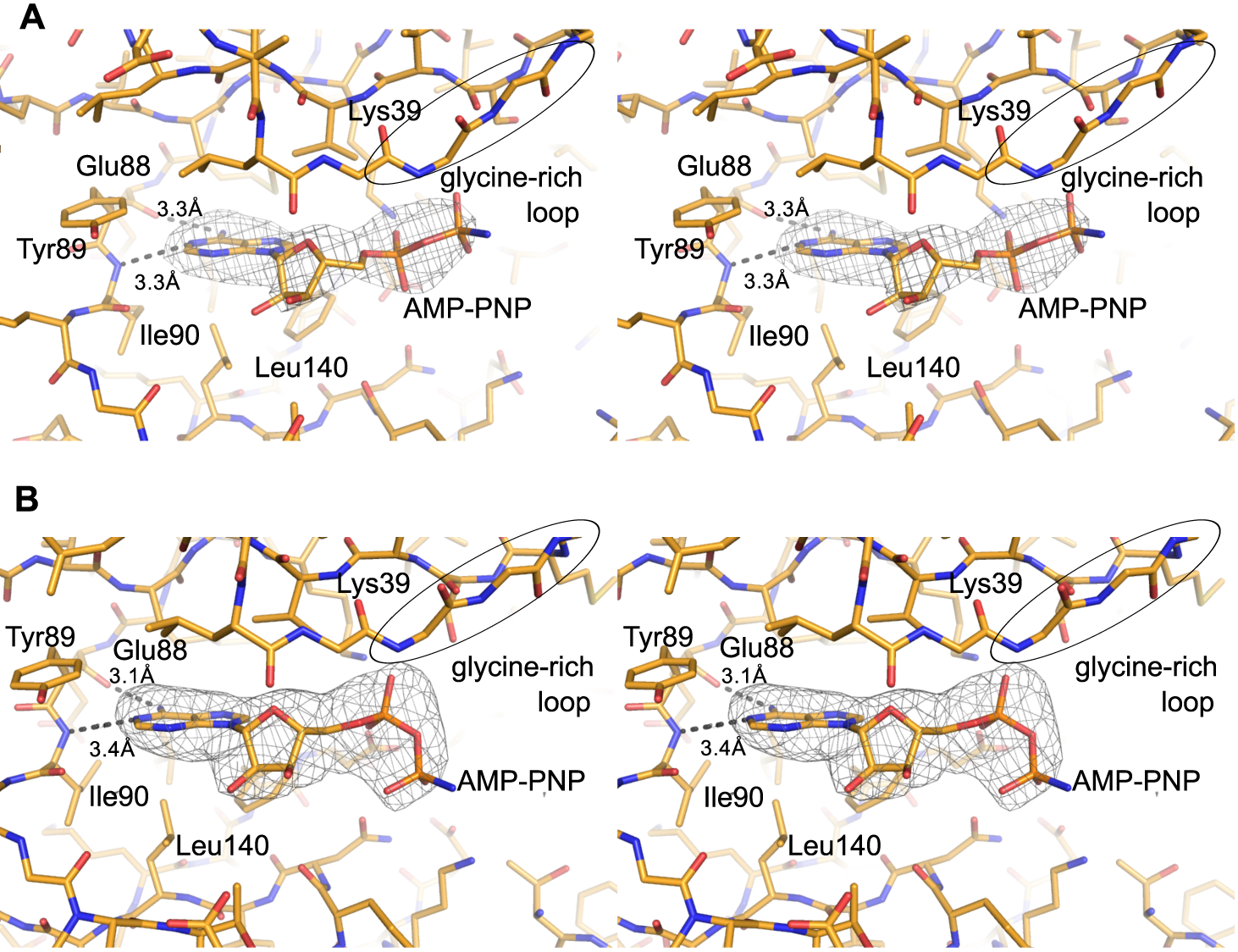

Supplement: Figure S3 — Stereo view into the AMP-PNP binding site. The depicted map is an omit map (Fobs-Fcalc) of the ligand, contoured at 3.0 σ and drawn with a radius of 5Å around AMP-PNP. Panels A and B show omit maps of the AMP-PNP bound to chains A and D, respectively. (TIF) [file pone.0039136.s003.tif]

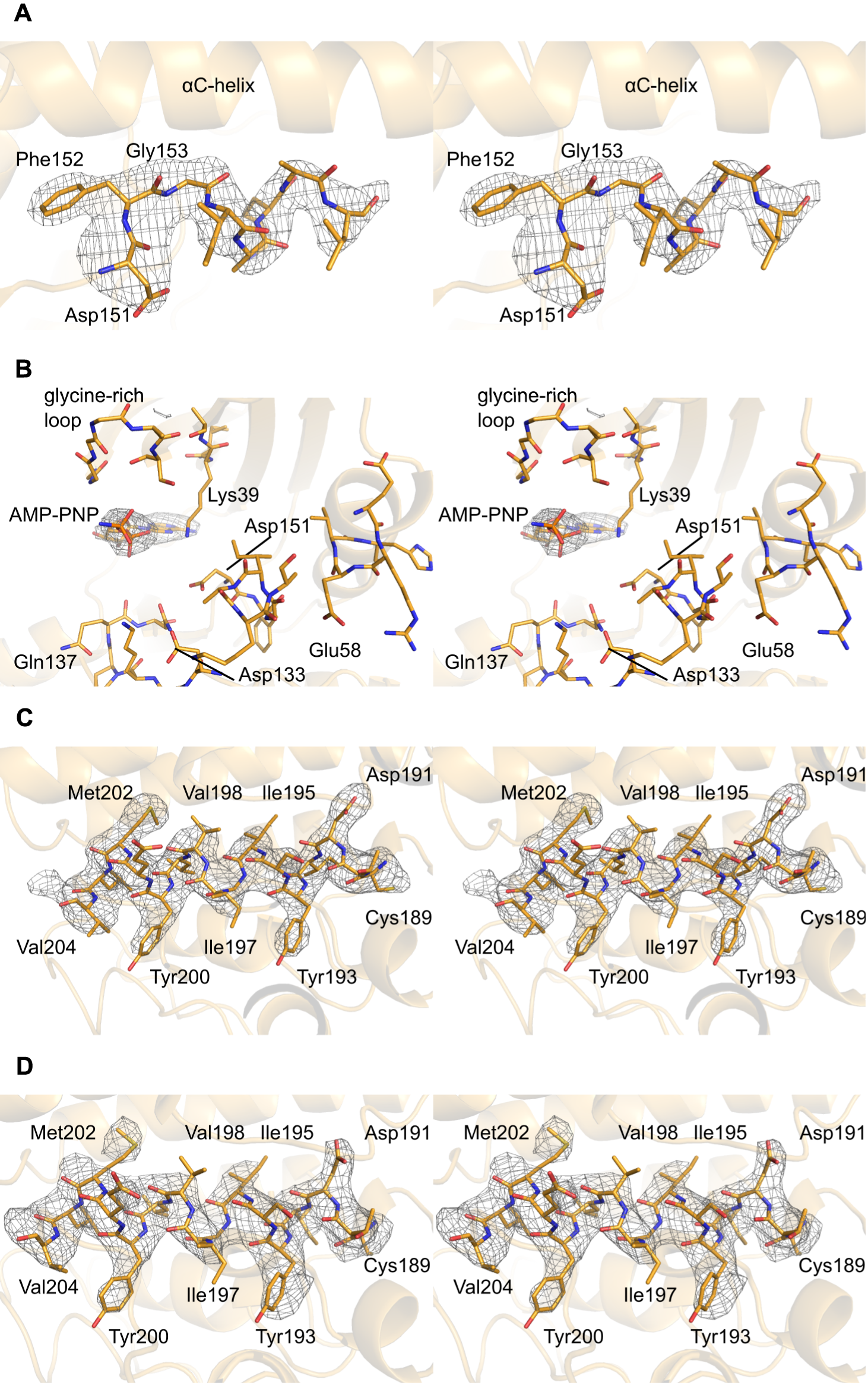

Supplement: Figure S4 — Stereo views of representative omit maps of PknBSA-KD. All panels show Fobs-Fcalc omit electron density maps contoured at 1.0 σ. In panel A the omit map for the DFG-motif and the inhibition helix is shown. Panel B shows the phosphate binding region. Panels C and D show the omit map for the αF-helix for chain A in panel C and chain D in panel D. Maps were drawn with radii of 3Å (panels A, C and D) and 8Å (panel B) around the depicted coordinates. The larger radius for panel B was chosen to show that no extra density that would account for a magnesium ion exists in the vicinity of the AMP-PNP ligand. (TIF) [file pone.0039136.s004.tif]

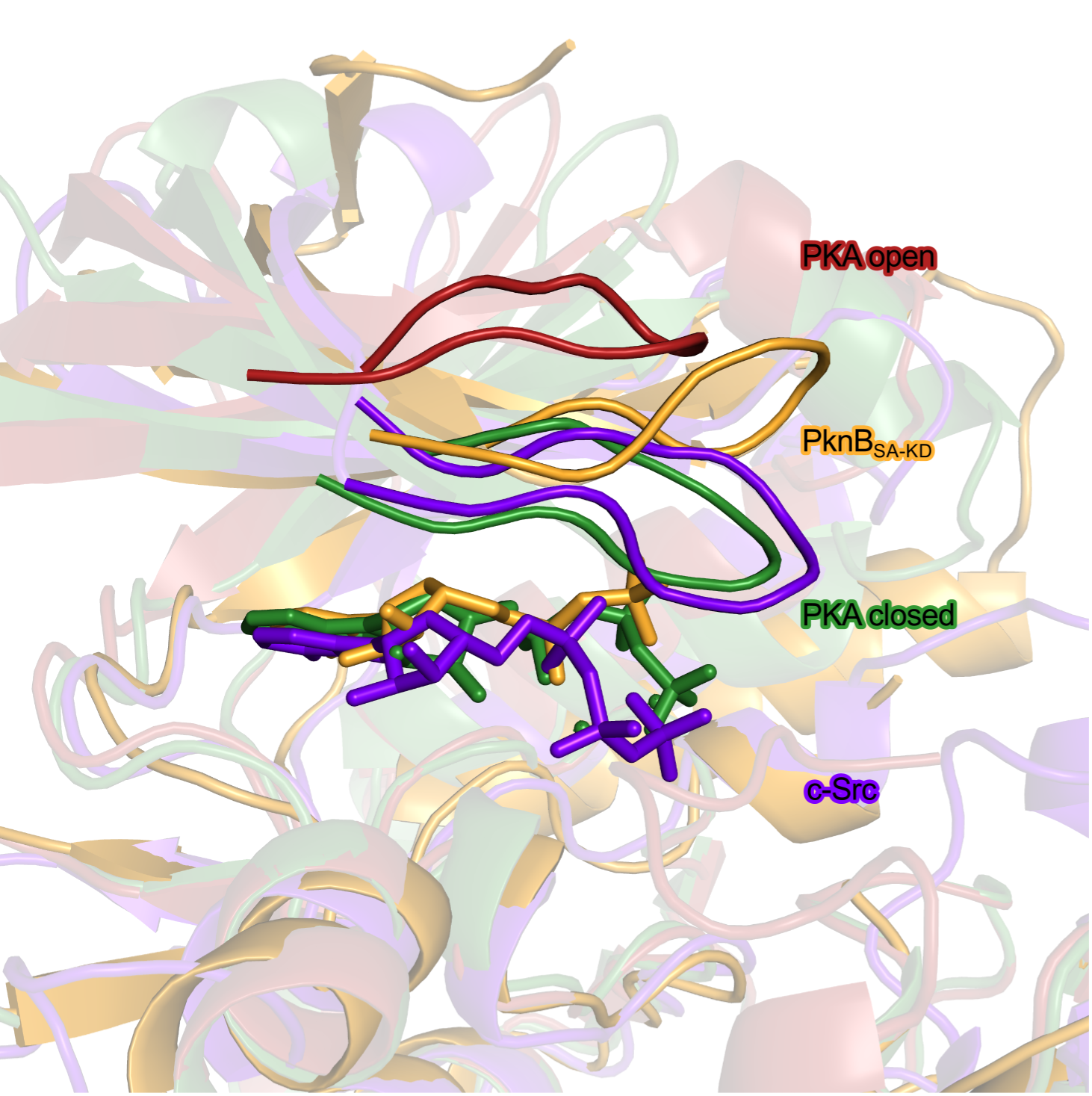

Supplement: Figure S5 — Orientation of the glycine-rich loop. All kinases were aligned with the C-lobe of PknBSA-KD (residues 100–250). The PknBSA-KD structure is drawn in orange. The closed PKA structure is shown in green (PDB ID: 1ATP [35]), and the open PKA structure is shown in red (PDB ID: 1CTP [34]). The kinase domain of c-Src (PDB ID: 2SRC [36]) is shown in purple. (TIF) [file pone.0039136.s005.tif]

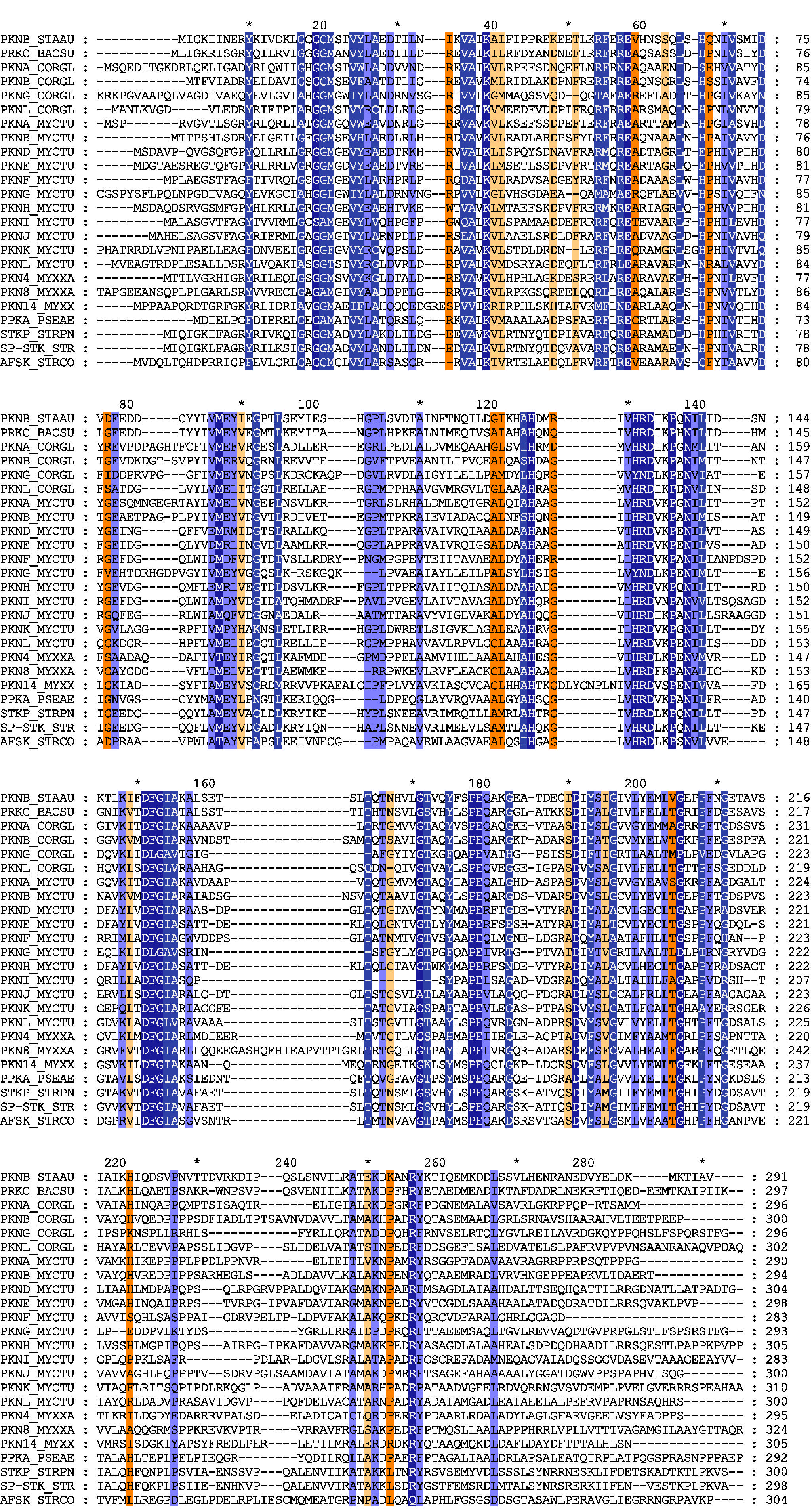

Supplement: Figure S6 — Alignment of selected kinases and analysis of conservation. The kinases were selected according to [10]. Five kinases were omitted due to lack of DFG-, SPE-, HRD-motif or the N-lobe. The color code is identical to that used in Fig 7. Blue indicates highly conserved residues (100–91% conservation in dark blue 88–74% in blue and 69–54% light blue). Residues highly conserved but different in PknBSA-KD are colored in orange (88–71% in orange, 69–54% in light orange). The selected kinases are (Uniprot-ID in parentheses): Staphylococcus aureus, PknB (Q7A5Z8); Bacillus subtilis, PrkC (O34507); Corynebacterium glutamicum, PknA (Q8NU97); Corynebacterium glutamicum, PknB (Q8NU98); Corynebacterium glutamicum, PknG (Q6M299); Corynebacterium glutamicum, PknL (Q6M3Q8); Mycobacterium tuberculosis, PknA (P65726); Mycobacterium tuberculosis, PknB (P0A5S4); Mycobacterium tuberculosis, PknD (O05871); Mycobacterium tuberculosis, PknE (P72003); Mycobacterium tuberculosis, PknF (P72003); Mycobacterium tuberculosis, PknG (P65728); Mycobacterium tuberculosis, PknH (Q11053); Mycobacterium tuberculosis, PknI (P65730); Mycobacterium tuberculosis, PknJ (P65732); Mycobacterium tuberculosis, PknK (P95078); Mycobacterium tuberculosis, PknL (O53510); Myxococcus xanthus, Pkn4 (Q95478); Myxococcus xanthus, Pkn8 (Q9XBP6); Myxococcus xanthus, Pkn14 (Q93NE3); Pseudomonas aeruginosa, PpkA (Q9I758); Streptococcus pneumonia, StkP (Q8KY50). (TIF) [file pone.0039136.s006.tif]

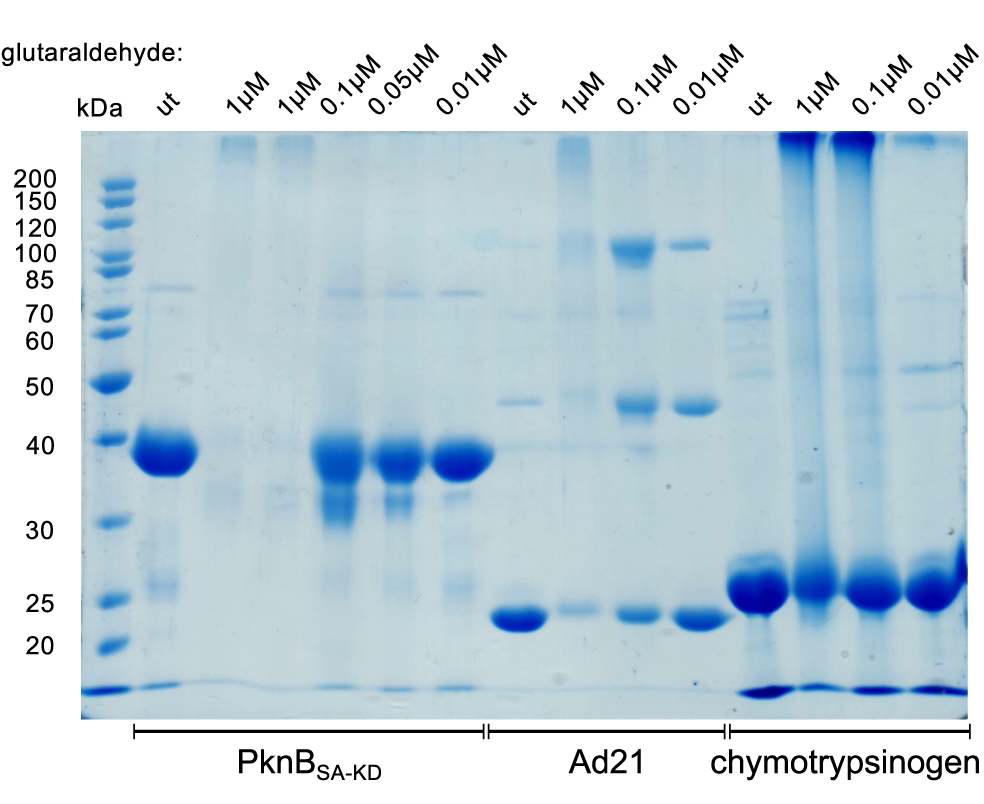

Supplement: Figure S7 — Chemical cross-linking of PknBSA-KD and controls with glutaraldehyde. Shown is an SDS-PAGE analysis of the crosslinking experiment. Untreated (ut) protein was loaded on the gel next to each protein as controls. The small bands in PknBSA-KD lanes indicate a weak impurity of PknBSA-KD at 80 kDa. (TIF) [file pone.0039136.s007.tif]
